# Coating with a Modular Bone Morphogenetic Peptide Promotes Healing of a Bone-Implant Gap in an Ovine Model

**DOI:** 10.1371/journal.pone.0050378

**Published:** 2012-11-21

**Authors:** Yan Lu, Jae Sung Lee, Brett Nemke, Ben K. Graf, Kevin Royalty, Richard Illgen, Ray Vanderby, Mark D. Markel, William L. Murphy

**Affiliations:** 1 Comparative Orthopaedic Research Laboratory, School of Veterinary Medicine, University of Wisconsin-Madison, Madison, Wisconson, United States of America; 2 Departments of Biomedical Engineering, University of Wisconsin, Madison, Wisconson, United States of America; 3 Pharmacology, University of Wisconsin, Madison, Wisconson, United States of America; 4 Orthopedics and Rehabilitation, University of Wisconsin, Madison, Wisconson, United States of America; University of California Davis, United States of America

## Abstract

Despite the potential for growth factor delivery strategies to promote orthopedic implant healing, there is a need for growth factor delivery methods that are controllable and amenable to clinical translation. We have developed a modular bone growth factor, herein termed “modular bone morphogenetic peptide (mBMP)”, which was designed to efficiently bind to the surface of orthopedic implants and also stimulate new bone formation. The purpose of this study was to coat a hydroxyapatite-titanium implant with mBMP and evaluate bone healing across a bone-implant gap in the sheep femoral condyle. The mBMP molecules efficiently bound to a hydroxyapatite-titanium implant and 64% of the initially bound mBMP molecules were released in a sustained manner over 28 days. The results demonstrated that the mBMP-coated implant group had significantly more mineralized bone filling in the implant-bone gap than the control group in C-arm computed tomography (DynaCT) scanning (25% more), histological (35% more) and microradiographic images (50% more). Push-out stiffness of the mBMP group was nearly 40% greater than that of control group whereas peak force did not show a significant difference. The results of this study demonstrated that mBMP coated on a hydroxyapatite-titanium implant stimulates new bone formation and may be useful to improve implant fixation in total joint arthroplasty applications.

## Introduction

Total joint replacement surgeries have been performed popularly because these surgeries can successfully relieve pain and improve functional outcomes. However, the failure rate of revision joint replacements is also dramatically higher than primary replacements due primarily to a challenging environment for new bone formation [1]. Therefore, there is a need to develop novel biomaterials to increase replacement success rate and reduce revision rate, and to promote successful bone healing after revision surgery. Besides improving surgery techniques [2], the key factor for successfully improving implant-bone healing in joint replacement is the ability to encourage new bone formation at the implant-native bone interface [3]. Therefore, several groups have begun to explore the use of pro-osteogenic growth factors to induce new bone formation on implant surfaces. For example, Sachse et al recently reported that the recombinant protein bone morphogenetic protein-2 (BMP-2) may foster bone healing on a titanium surface in a sheep model [4]. Further, Lamberg et al stated that transforming growth factor-β1 (TGF-β1) and insulin-like growth factor-1 (IGF-1) enhanced the mechanical fixation and osseointegration of titanium implants in cancellous bone in a dog model [5].

Collectively, previous studies demonstrate the potential for growth factor delivery strategies to promote orthopedic implant healing. However, translation of growth factor delivery to clinical applications is plagued by significant challenges [6]. First, growth factor delivery strategies typically do not use carriers with optimal physical properties. The common strategy instead involves designing a growth factor “carrier”, then combining it with a standard orthopedic device with appropriate physical properties. Notable examples include recombinant protein-loaded collagen carriers combined with titanium cages [7], [8] or other metallic hardware [9]. Innovative biomaterials for controlled growth factor release [10], ranging from polymer scaffolds to injectable micro- and nano-particles, also typically require combination with a structural device. In addition, existing growth factor carriers contain supraphysiologic doses delivered over relatively short timescales, which has recently led to well-documented side effects including edema [11], [12], heterotopic bone formation [13], retrograde ejaculation [14], and potentially increased cancer risk [15].

Bone morphogenetic proteins (BMPs) are the key cytokines in bone formation and repair. A possible strategy to utilize BMPs’ activity in clinical applications is to enhance the activity of autologous BMPs. BMP-2 incorporated into biomimetic calcium phosphate coatings has been demonstrated to be capable of inducing bone formation at an ectopic site and sustaining osteogenic activity for a considerable period of time [16], [17].

We hypothesized that a modular bone morphogenetic peptide (mBMP) could be used to “dip-coat” the surface of an orthopedic implant, which in turn would be capable of promoting implant-native bone healing. Specifically, we synthesized a peptide with two functional units: i) an osteocalcin-inspired hydroxyapatite (HAP)-binding sequence; and ii) a peptide sequence previously shown to mimic some of the pro-osteogenic properties of the protein BMP-2 [18], [19]. We then would perform experiments to determine whether this peptide could “dip-coat” the surface of an orthopedic implant and, in turn, promote bone healing across a well-defined bone gap in a sheep model. The bone-implant gap in the current study was designed into the implant to more effectively mimic the challenging environment that is typically present during revision joint arthroplasty. We hypothesized that an HAP-titanium implant dip-coated with mBMP inserted in cancellous bone would result in improved mechanical implant fixation and induce more bone ingrowth across the defined gap zone.

## Materials and Methods

### HAP-titanium Implants

**Figure 1:**
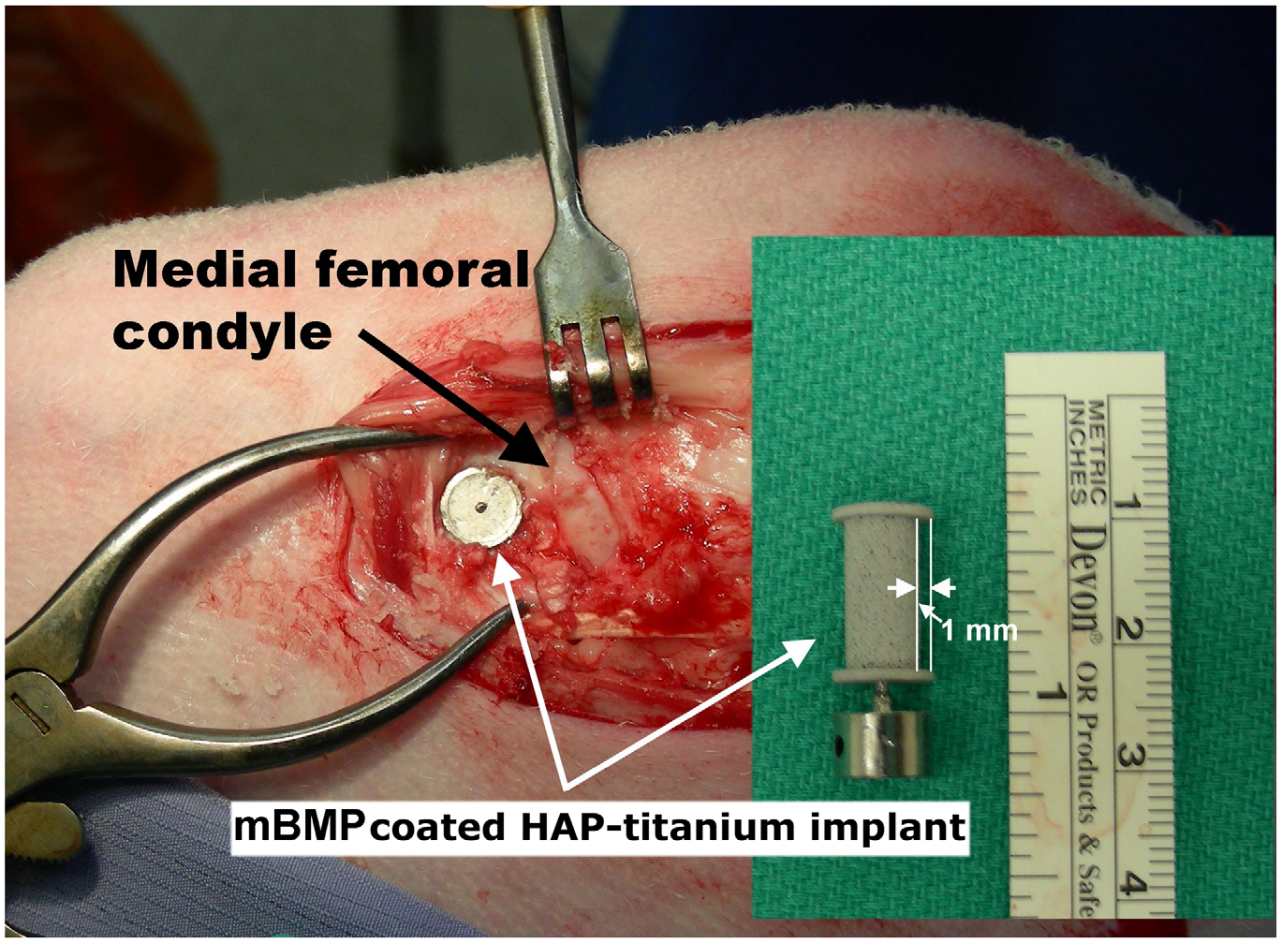
Image of mBMP coated HAP-titanium implant and surgical implantation in sheep medial femoral condyle.

Custom implants (diameter: 8 mm, HAP coated length: 14 mm. Smith and Nephew Corp. Andover, MA) were “porous coated”, meaning that they incorporate a porous titanium surface that is plasma spray bound with hydroxyapatite. In addition, the implants were designed to include a 1 mm gap all around the perimeter of the cylindrical implants, so that there was a well-defined gap between the native bone and the implant surface (Fig. 1).

### Peptide Binding and Release from HAP-titanium Implant

Modular bone morphogenetic peptide (Amino acid sequence of mBMP; KIPKASSVPTELSAISTLYLAAAAγEPRRγEVAγEL) was synthesized using standard Fmoc solid phase peptide synthesis, as reported previously [20], [21]. Carboxyfluorescein (Molecular Probes Inc., Eugene, OR) was used to label mBMP to monitor its release from the implants into simulated body fluid (SBF; 136.8 mM NaCl, 3 mM KCl, 0.5 mM Na_2_SO_4_, 1.5 mM MgCl_2_, 4.2 mM NaHCO_3_, 2.5 mM CaCl_2_, 1.0 mM K_2_HPO_4_, 50 mM Tris base). First, fluorescently labeled mBMP was bound on implants by incubating each implant in peptide solution (4 mL, 1.9 mM) for 4 hours at 37°C. The mBMP-bound implant was then incubated in 3 mL of SBF solution at 37°C in a static condition to examine the release behavior of peptide. At each time point (day 1, 2, 3, 6, 9, 13, 18, 22 and 28), the fluorescence intensity of release medium was measured and the release medium was replaced with a fresh one. After 4-week release, the mBMP remaining on implant was retrieved by incubating in 0.5 N hydrochloric acid solution overnight. The amount of mBMP was quantified using microBCA (Thermo Fisher Scientific Inc., Rockford, IL) after neutralizing with sodium hydroxide solution, and the amount of initial binding was calculated by adding to that of released peptide.

### mBMP Coating of Implants for in vivo Studies

Each experimental HAP-titanium implant was incubated in 4 mL of mBMP solution (1.9 mM) 4 hours at 37°C. The mBMP solution was sterilized by passage through sterile 0.22 µm filters. All incubation procedures were performed in aseptic conditions.

### In vivo Experimental Design

Twelve mature female sheep, ranging in age from 3.5 to 5 years and weighing between 70 to 110 kg (82.4±5.6 kg: mean±SD) were utilized in the study. All experimental protocols were approved by the Institutional Animal Use and Care Committee of University of Wisconsin-Madison.

In each of the 12 sheep, one of the stifles was randomized (block design) to receive an mBMP-coated HAP-titanium implant and the other (contralateral) received an un-treated implant (n = 12/treatment). The sheep were euthanized at 4 weeks after surgery. 8 sheep (16 stifles, 8/group) were subjected to C-arm computed tomography (DynaCT) and biomechanical testing, 4 sheep (8 stifles, 4/group) were used for histologic analysis.

### Surgical Procedure

Surgery was carried out under general anesthesia with isoflurane and oxygen inhalation via endotracheal intubation. Procaine Penicillin G (5 mls) was administered via muscle pre-operatively and an additional dose (5 mls) post-operatively. An 8 cm medial incision was made along the anterior edge of medial collateral ligament in one randomly selected stifle. The medial portion of medial condyle was exposed. An 8 mm diameter hole (14 mm deep) was drilled from the medial surface of the condyle near to collateral ligament origin by an 8 mm cannulated drill bit 5 mm proximal to the joint line taking care to avoid the joint. An mBMP-coated HAP-titanium implant was inserted in the hole (Fig. 1). Then the surrounding soft tissue was sutured and the implant was covered. In the contralateral stifle, the identical procedure of exposure was performed with the un-treated implant in the medial condyle. The incision was lavaged by sterile saline. The deep fascia, subcutaneous tissue and skin incision were closed as routine.

After 4 weeks, all sheep were euthanized for biomechanical, DynaCT and histological analyses.

### Dyna CT Scan

At the same day of sacrifice, the medial femoral condyles were cut into uniform bone blocks (3.0×3.0×2.0 cm^3^) and imaged using a Siemens Artis Zeego (Siemens Healthcare, Erlangen, Germany) C-arm computed tomography (DynaCT), which has a wide angle cone-beam x-ray tube and 40 cm × 30 cm flat-panel detector integrated with a robotic C-arm gantry. The acquisition parameters for DynaCT were as follows: 86 kV tube voltage, automatic tube current ranging from 70 to 125 mA, a 20 s rotation time (total of 496 projections over 200 degree sweep angle). The projection images were acquired in a 2 × 2 binning mode, providing a 154 µm detector element resolution. Acquired images were then transferred to a dedicated post-processing workstation (*syngo* X-Workplace, Siemens Healthcare, Erlangen, Germany) where a volume data set was reconstructed to a 512×512 matrix and 306–328 slices with an isotropic voxel size of 140 µm using a sharp bone kernel for visualization of the bony structure.

The Dyna CT image of each specimen was analyzed with Image J software (National Institutes of Health, Bethesda, MA). The area of bone ingrowth in the gap between the implant and hosting bone was calculated and compared between mBMP-coated and control groups.

### Biomechanical Testing

After DynaCT scan, the femoral condylar bone blocks were used for push out mechanical testing. The specimen was placed in a MTS 858 BIONIX Test System (MTS Systems Corp., Eden Prairie, MN) with a 1500 lb load cell. The titanium implant in the specimen was precisely aligned with a pushing shaft. A linear extensometer was attached to measure the displacement of the implant. The displacement rate was 5.0 mm/min as previously described [22]. A 50 N preload was applied. Peak force at failure (N) and stiffness (N/mm) were measured and recorded. Peak force was determined from the load-displacement curve. Stiffness was calculated by measuring the slope from the linear portion of the curve.

### Histological Analysis

The remaining 8 condyles in 4 sheep were used for histological analysis. The specimens were fixed in neutral buffered 10% formalin for 1 week. The medial condyle was sectioned sagitally into 3 equal sections crossing the implant axis. The undecalcified specimens were embedded in polymethylmethacrylate (PMMA), sectioned (100 µm) and stained with Goldner’s Trichrome for the evaluation of implant to bone healing. The sections from each specimen were examined by three senior researchers blinded to group assignments. Fine detail contact microradiography (Hewlett-Packard Faxitron, McMinnville, OR. USA) combined with vacuum technique was also performed on 3 sections of each specimen as previously described. [23] The 3 sections were defined as “outer” (near to cortex), “middle”, and “inner” (farther to cortex). Bone density in the implant-bone gap region of each 3 section levels and total bone density of 3 sections [(sum of bone-occupied area of “inner”, “middle” and “outer” groups)/(sum of area of interest of “inner”, “middle” and “outer” groups)*100] were quantified using Image J software for both histological and microradiographical images.

### Statistical Analysis

The Shapiro-Wilk Test was first used to determine the normal distribution (Gaussian distribution) of histological, microradiographic, DynaCT and mechanical testing data sets. If the data sets were normally distributed, the Student’s Paired t-test was used for statistical analysis for histology, microradiography, DynaCT and mechanical testing data between the two treatment groups. If the data sets were detected to be not normally distributed, the Mann-whitney U test was used to compare these differences. Differences were considered to be significant at a probability level of 95% (p<0.05). All statistical analyses were performed with a commercially available software program (SAS Version 8e, SAS Institute Inc., Cary, NC).

## Results

### In Vitro

**Figure 2:**
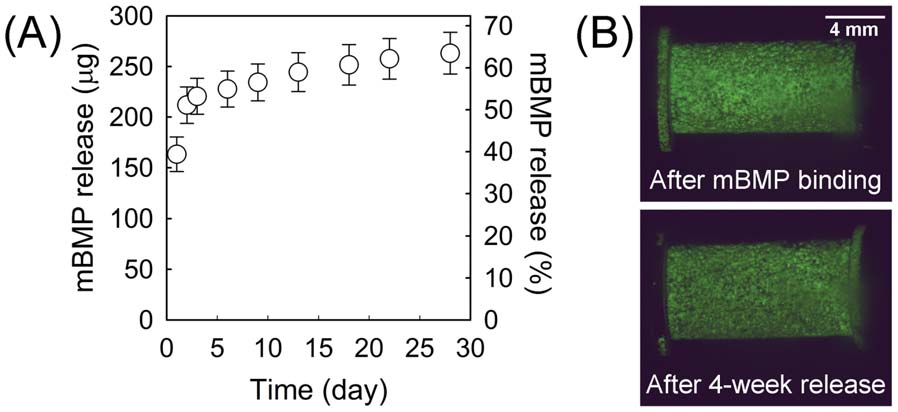
Images of release profile of mBMP in vitro. (A) Cumulative release of mBMP from HAP-titanium implant in SBF is over 60% at 4 weeks. (B) Fluorescent images of an implant after incorporation with fluorescently labeled mBMP (top) and after 4-week incubation in simulated body fluid (SBF) (bottom).

The amount of mBMP initially bound to a HAP-titanium implant was 414.4±22.8 µg. The release profile of mBMP in SBF showed large initial release 51.1±2.9% of initially bound mBMP during first two days (Fig. 2A). Afterwards, the mBMP was released in a nearly linear fashion for 4 weeks with total release of 263.1±20.7 µg (63.4±1.8% of initially bound mBMP). As evidenced in Figure 2B, a significant amount of mBMP molecules still resides on HAP-implant surface after 28-day release.

### In Vivo

No postoperative complications were seen and all sheep were fully weight-bearing at the same day after surgery. No signs of infection were observed at the time of euthanasia.

### CT Results

**Figure 3:**
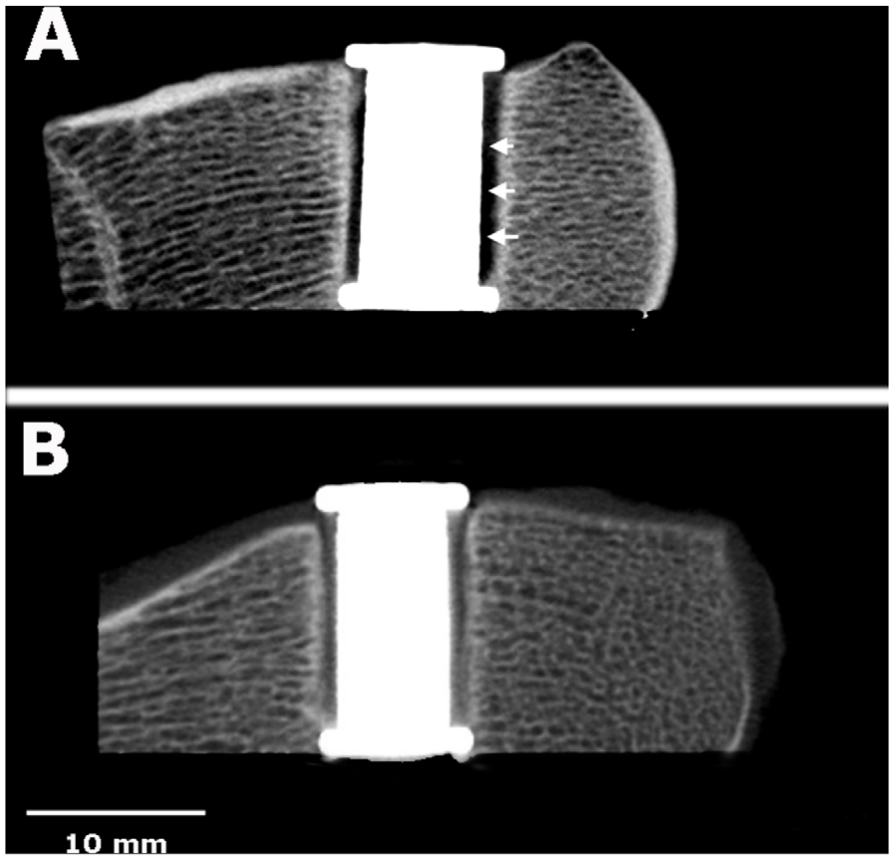
Images of Dyna CT results between control and mBMP coated implants. A: Control, DynaCT demonstrating that gap between the implant and host bone was still visible (white arrows). B: The gap between mBMP coated HAP-implant and host bone was filled with high density mineralized tissue.

DynaCT analysis demonstrated that the mBMP-treated group (4.7±2.0 mm^2^) had a significantly higher density of mineralized bone tissue filling the 1 millimeter bone-implant gap than the control group (3.7±2.0 mm^2^) (p<0.05) (Fig. 3).

### Histological Results

**Figure 4:**
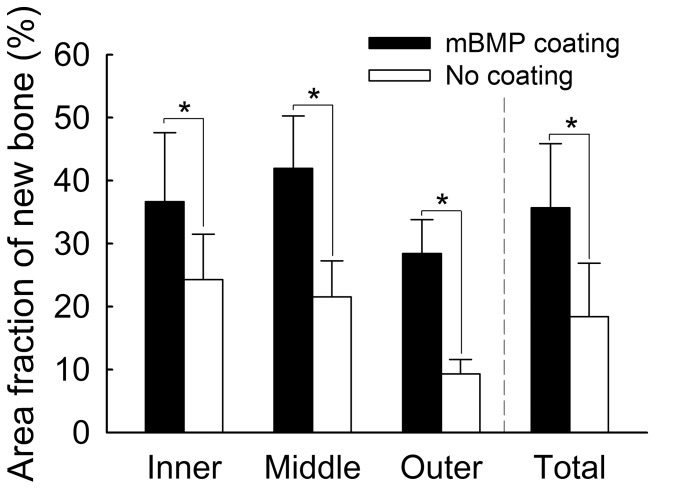
Microradiographic calculation of new bone formation in the gap demonstrating mBMP group had significantly more new bone ingrowth than non-mBMP group at 3 levels of the implant and in total amount. “*” means significant difference between treatments (p<0.05).

**Figure 5:**
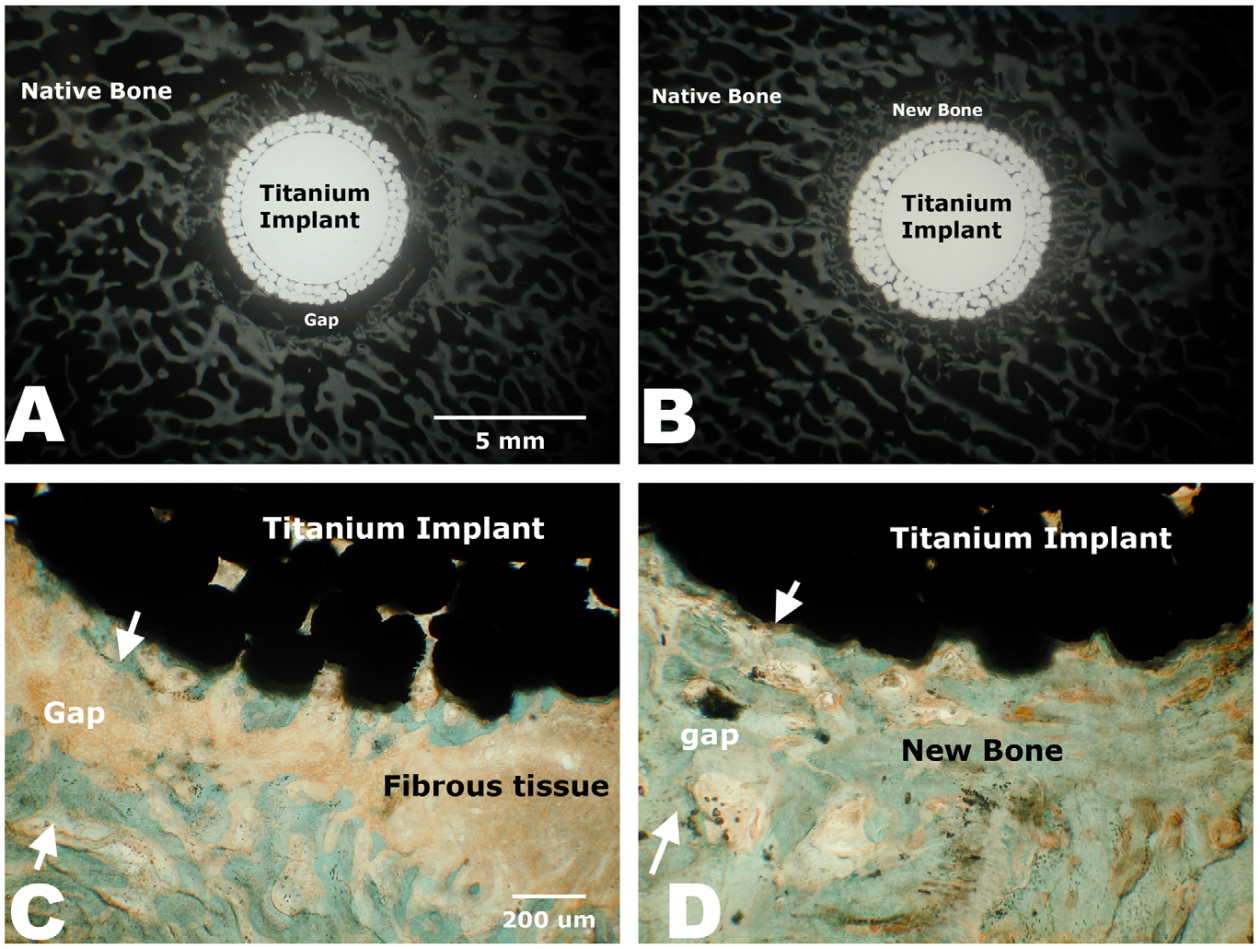
High detailed radiograph (A, B) and histologic section (C, D) of non-mBMP coated HAP-implants (A, C) and mBMP-coated implants (B, D). In C and D, areas between white arrows highlighted original 1-mm gap between the implant and host bone.

**Figure 6:**
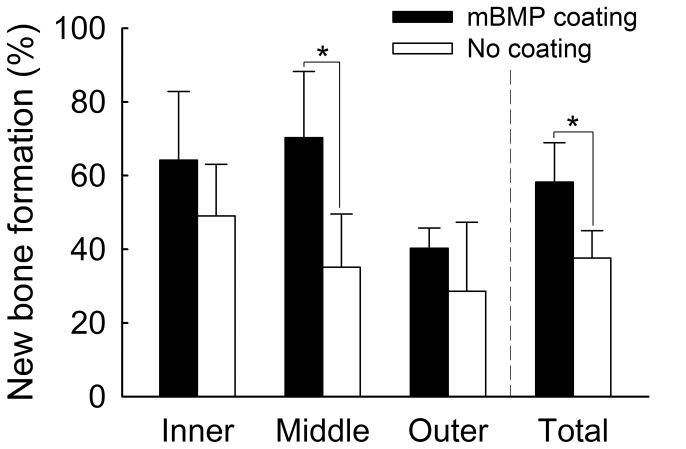
Histologic calculation of new bone formation in the implant-bone gap on the Goldner’s trichrome staining slides demonstrated that middle section and total 3 sections of mBMP coated implant had significantly more new bone formation compared to control. “*” means significant difference between treatments (p<0.05).

Microradiographical data analyses showed the mBMP-coated HAP-titanium implants had significantly greater area of higher bone density in the bone-implant gaps compared to the controls (p<0.05) (Fig. 4). This increased bone formation was observed throughout the bone-implant interface - at outer, middle, and inner section levels. Microradiographical images also demonstrated that the control group had significantly less new bone formation in the bone-implant gap than the mBMP-coated implant group (Fig. 5 A and B). Histological analysis further demonstrated significantly greater new bone formation in the bone-implant gap in the mBMP-coated implant conditions (Fig. 5 C and D). Specifically, calculation of new bone formation in Goldner’s trichrome stained images demonstrated a significant increase in new bone formation in the middle section and in the inner/middle/outer section levels combined for mBMP coated implants when compared to controls (p<0.05) (Fig. 6).

### Mechanical Testing Results

The results of mechanical push-out testing demonstrated that the stiffness of the mBMP-treated group (2157±651.9 N/mm) was significantly greater than that of control group (1545±480.5 N/mm) (p<0.05), whereas there was no significant difference in peak force between the mBMP-treated group (1022±371.4 N) and the control group (682.2±269.3 N) groups (p>0.05).

## Discussion

The mBMP peptides were efficiently bound to a HAP-titanium implant and released over 28 days in simulated body fluid. *In vivo* results of this study demonstrated that mBMP-coated HAP-titanium implant increased implant fixation and stimulated bone formation in the gap between implant and surrounding host cancellous bone after 4 weeks post-surgery compared to the control.

mBMP release kinetics showed a substantial initial release of over 50% during the initial 2 days, followed by minimal release kinetics over more than 28 days. These distinct regimes of mBMP release can be attributed to an initial release of poorly bound peptide due to surface saturation, followed by slower release of mBMP that was strongly bound to the hydroxyapatite surface. In particular, the solution concentration used for mBMP binding here (1.9 mM) was much higher than the concentration previously shown to saturate binding to a hydroxyapatite surface [20], [24], which likely explains the rapid initial release kinetics. The minimal mBMP release after the first two days is indicative of the high mBMP-implant binding affinity, as demonstrated previously [20]. The observation that nearly 40% of the initially bound mBMP remained on the implant surface after 4 week incubation is potentially interesting in the context of contemporary growth factor delivery strategies. Rapid and poorly-localized bone growth factor delivery can lead to significant clinical side effects, as demonstrated by recent clinical analyses of BMP-2-releasing implants [13], [14], [25]. Although further studies will be needed to demonstrate control over mBMP dosage on the implant surface, our previous studies have demonstrated that mBMP dosage can be controlled by simply varying the mBMP concentration in solution during implant dip-coating [20], [21], [24].

Sheep were selected for this study because they provide similar bone density and weight to humans, and they have been widely used for evaluation implant-bone healing in previous studies [4], [26]–[29]. The sheep femoral condyle was particularly used as a model in this study because it is rich in high quality cancellous bone with ample blood supply, which is suitable for an evaluation of early implant bone healing [22]. In addition, it is also representative of the bony fixation regions, such as proximal femoral prostheses in humans. The gap size (1.0 mm) selected for this study was defined as critical size under non-loading condition based on a previous study [22]. The sacrifice time at 4 weeks post-surgery was selected for monitoring early stage of bone ingrowth in the gap between the titanium implant and host bone. Several previous studies have used the same time point to evaluate bone ingrowth in bone-implant gaps in animal models [22], [30], [31].

DynaCT, histological and microradiographical results of this study demonstrated that significantly higher density tissue and new bone formation was present in the gap between mBMP-coated HAP-titanium implant and condylar bone compared to control. An interesting observation here was that endochondral ossification with the formation of cartilaginous tissues was not observed at 4 weeks post-surgery. Instead, it appeared that direct ossification of mesenchymal tissue as intramembraneous ossification was taking place, as reported in a previous study [4].

New bone formation in the gap between the implant and native host bone can also increase rigidity and fixation of an implant, which has been validated in the current study. The push-out stiffness of mBMP coated HAP-titanium implant group was significantly greater than that of control although the peak force did not reveal a statistically significant difference. Few studies to date have performed mechanical push-out testing on HAP-titanium implants treated with biologic molecules. Elmengaard et al evaluated the effects of an Arg-Gly-Asp (RGD) peptide coating on tissue integration and titanium implant fixation across a bone-implant gap in dogs [22]. At 4 weeks post-surgery, it was reported that RGD-coated implants had significantly greater push-out stiffness compared to controls, whereas the peak strength was not significant different [22]. Sachse et al performed a study to evaluate HAP-titanium implants in sheep and reported that implants treated with BMP-2 displayed 50% higher pull-out force than controls at 20 weeks post-surgery [4]. Relative to these previous studies, the current study indicates that the modular peptide mBMP may also be useful in total joint arthoplasty, and other orthopedic implant applications. In addition, the ability of mBMP to bind with high efficiency to hydroxyapatite-containing implants may be particularly advantageous, as our previous studies have shown that efficient binding results in controllable dosages and localized release [20], [21], [24], [32]. In addition, we have recently shown that mBMP can be modified to control the release rate from hydroxyapatite substrates [20], [21], [32]. Finally, recent studies indicate that the approach described here can be applied to other biologically active molecules, such as vascular endothelial growth factor (VEGF) [33]. Collectively, these studies suggest that the current approach may be useful in a range of orthopedic implant applications.

It is noteworthy that specific aspects of this study were designed for relevance to potential clinical applications of these biomaterials. First, the bone-implant gap size of implant selected for this study (1 mm) was a critical size, which may be larger than the gap in actual human clinical applications, but may represent a challenging scenario for bone-implant healing in revision joint arthroplasty. Second, the four week evaluation time is a relatively early time point to characterize new bone formation, and may provide particular insights into the degree of accelerated bone formation. A later time point could provide insight into the ability of mBMP to promote stable long-term implant fixation, but would not indicate an ability to promote the rapid fixation that is needed. The purpose of this study was to characterize a challenging bone-implant gap and an early time point, to more directly represent the challenging environment present during revision arthroplasty, and the need to accelerate fixation and avoid development of fibrous tissue that can lead to pain and implant instability.

### Conclusions

The mBMP peptides were efficiently bound to a HAP-titanium implant and released over 28 days in a sustained manner *in vitro*. *In vivo* results demonstrated that mBMP-coated HAP-titanium implants increased implant fixation and stimulated bone formation in a well-defined, critical gap between the implant and surrounding host cancellous bone after 4 weeks post-surgery compared to untreated HAP-titanium implants. This technique may be beneficial for human clinical applications, such as mBMP-coating on the stem of prosthesis, to enhance new bone formation at early stages.
